# Assistance for the Prescription of Nutritional Support Must Be Required in Nonexperienced Nutritional Teams

**DOI:** 10.1155/2013/450469

**Published:** 2013-12-17

**Authors:** Mehdi Ouaïssi, Philippe Grandval, Diane Mege, Anamaria Nedelcu, Gaëlle Hautefeuille, Frédéric Vanhoeve, Bernard Sastre, Igor Sielezneff, Jacques di Costanzo

**Affiliations:** ^1^Department of Digestive Surgery, Timone Hospital, 13385 Marseille, France; ^2^Aix Marseille University, UMR 911, Campus Santé Timone, 13385 Marseille, France; ^3^Department of Gastroenterology, Timone Hospital, 13385 Marseille, France; ^4^Department of Pediatric Surgery, Hospital University of Geneva, 1205 Geneva, Switzerland; ^5^Synergie Médical, 380 Avenue des Templiers, Aubagne, France

## Abstract

The aim of the study was to determine the current practices of nutritional support among hospitalized patients in nonspecialized hospital departments. *Materials and Methods*. During an observation period of 2 months, a surgeon and a gastroenterologist designated in each of the two departments concerned, not “specialized” in nutritional assistance, have treated patients in which nutritional support seemed necessary. Assessing the degree of malnutrition of the patient, the therapeutic decision and the type of product prescribed by the doctors were secondarily compared to the proposals of a structured computer program according to the criteria and standards established by the institutions currently recognized. *Results*. The study included 120 patients bearing a surgical disease in 86.7% of cases and 10% of medical cases. 50% of the patients had cancer. Nutritional status was correctly evaluated in 38.3% by the initial doctors' diagnosis—consistent with the software's evaluation. The strategy of nutrition was concordant with the proposals of the software in 79.2% of cases. *Conclusions*. Despite an erroneous assessment of the nutritional status in more than two-thirds of cases the strategy of nutritional management was correct in 80% of cases. Malnutrition and its consequences can be prevented in nonexperienced nutritional teams by adequate nutritional support strategies coming from modern techniques including computerized programs.

## 1. Introduction

About 40% of the patients admitted to hospital show different degrees of malnutrition [1]. If this deficiency is not identified and correctly treated in time, it can eventually become more severe and induce a significant increase in the morbidity/mortality rate with a subsequent increase in the length of the hospital stay [2, 3]. Currently, few studies allow us to evaluate the effects upon these parameters, of appropriate nutritional support during hospital stay [4, 5]. This is partly due to the fact that in nonspecialized departments, without an experienced nutritional team, the nutritional prescriptions usually remain poorly adapted.

The aim of the study was to determine the current practices of nutritional support among hospitalized patients in nonspecialized hospital departments.

## 2. Material and Methods

### 2.1. Hospital Departments

Two departments were selected at the CHU of Marseille: gastroenterology and visceral surgery. During a 2-month observation period, a gastroenterologist and a visceral surgeon were, respectively, assigned to each department, both nonspecialists in the field of nutrition, and they prescribed nutritional support according to their usual criteria to patients in whom nutritional support seemed to be indicated/necessary.

### 2.2. Definitions

Initially, the doctors recorded the weight and the loss of weight (normal, mild, or severe malnutrition). Digestive pathology and treatment (type of surgery) were prospectively collected in the software. Malnutrition treatment (enteral or parenteral nutrition, nutritional assistance) and nutritional cocktails data (volume, calories, calorie-nitrogen ratio, glucose-lipid ratio, nitrogen, and electrolytes) were equally recorded.

Once the weight, height, and loss of weight expressed as a % of usual weight related to the duration of weight loss were registered, the Body Mass Index was automatically obtained allowing for the calculation of the Nutritional Risk Index and subsequently the risk of malnutrition. According to these data, the software was able to calculate the patient's level of energetic needs (Total Energy Expenditure, calculated from Resting Energy Expenditure, resulting from Harris and Benedict formula, corrected by a coefficient ranging from 1.2 to 2, according to the activity of the patient and severity of the disease). The needs—water, electrolytes, vitamins, and trace elements—were finally completed.

The software, designed according to the recent proposals of the “Agence Nationale d'Accréditation et d'Evaluation en Santé” and within the “Programme National Nutrition et Santé” [2–6], was used to determine prescription proposition with an appropriate nutritional mixture/product.

Having recorded the data, the software proposed the evaluation of the nutritional status of the patient and the doctor was asked to follow the indications of the algorithm. Severely malnourished patients were defined by patients who cannot take a diet covering at least 60% of their nutritional needs within 1 week after surgery; patients with an early postoperative complication (sepsis, respiratory, or renal insufficiency, acute fistula, acute pancreatitis).

According to the results of the NRI, the software proposed, in cases of mild malnutrition, to seek the advice of a specialized nutritional team in order to prescribe nutritional complements or a balanced diet. In cases of severe malnutrition, the software proposed, depending on the functional state of the gastrointestinal tract, enteral nutrition or parenteral nutritional support or total parenteral nutrition.

After 5 minutes necessary to complete the process, the software proposed the most appropriate nutritional mixture according to the patient's needs. Finally, the software allowed the doctor either to conserve his initial evaluation (nutritional assessment, strategy of nutritional support, and nutritional mixture) or to modify some or all of the parameters. A variation of more than 20% from the calculated ideal values for any component of the nutritional mixture was systematically announced by a visual signal.

## 3. Results (Figure 1)

### 3.1. 120 Patients Were Included in the Study (Table 1)

Eighty six point seven % of them were hospitalized in the visceral surgery department and 14% in the medical gastroenterological department; 3.3% of the patients needed radiochemotherapy. Nutritional support was needed for surgical procedures in 55% of the cases; in 14% the diseases affecting the GI tract implied extended lesions (malabsorption, Crohn's disease, and chronic intestinal pseudo-obstruction) and short bowel syndrome in 5% of cases and 26% of the cases were uncategorized. 50% of the patients had cancer.

### 3.2. Nutritional Status

According to the software data, 100% of the patients selected demonstrated severe malnutrition. In 38.3% of these cases the initial diagnosis given by doctors was in agreement/consistent with that proposed by the software; in 38.3% of cases the doctors detected moderate malnutrition and in 23.4% of cases an absence of malnutrition. In 57.5% of cases the doctors maintained their initial diagnosis; the propositions offered by the software were adopted in 42.5% of cases.

### 3.3. Nutritional Support

The modalities/types of nutritional support, including enteral or parenteral assistance or total parenteral nutrition, proposed by the doctors were similar to those of the software in 79.2% of cases; the initial personal decisions of the doctors were unchanged in 16.7% of cases; those of the software were taken into account in 4.1% of cases.

### 3.4. Prescriptions

The doctors' prescriptions of nutritional mixtures/cocktail were matching those proposed by the software in 17.5% of cases; those of the software were taken into account in 50.8% of cases; initial personal prescriptions were unchanged in 31.7% of cases.

### 3.5. Analysis of the Subgroups of Patients

When subgroups of patients were considered, in cancer patients, software and doctors agreed in the nutritional status assessment in 38.3% of cases, in modalities of nutritional support in 80% of cases, and in composition of nutritional mixture in 15% of cases; in noncancer patients, accordance was successive and in the same order as 36.7%, 78.3%, and 21.7% of cases.

## 4. Comments

Strategic decisions of nutritional support are obviously randomly taken in University Hospitals in spite of the fact that doctors routinely treat malnourished patients with severe diseases. Nevertheless, even if malnutrition was misevaluated by doctors in more than 2/3 of cases, the need for nutritional support was confirmed by the software in 100% of cases. However, the strategy of nutritional support proposed by the doctors was adequate in nearly 80% of cases while nutritional mixture prescriptions were adequate in almost 15% of cases. In spite of an obvious inconsistency within the global strategy of nutritional support and inaccuracies in malnutrition assessment leading to inadequate prescriptions of nutritional mixtures, in most of the cases, doctors were able to correctly indicate the nutritional strategy. On the other hand, the fact that doctors modified their initial nutritional mixture prescription according to that proposed by software while they maintained their initial diagnosis and strategies in almost 4% of the cases seems to be a reassuring argument. This reveals that doctors might be aware of their lack of training on nutritional topics. The overall data are quite comparable either in cancer or in noncancer patients. This leads to the conclusions that either doctors need specialized training in nutrition or interventional teams could intercede with supposed malnourished patients. The proposed software, leading to appropriate therapeutic decisions in most of the cases, could resolve these difficulties.

In fact, the use of the proposed software could contribute to optimizing the strategy of nutritional support in hospitalized patients and subsequently reduce postoperative complications and mortality rates and duration of hospital stay. This needs to be demonstrated by further prospective studies using the software, which would allow for standardized prescriptions. Furthermore, beyond the medical and economic consequences, the training capacities of the software for practitioners could be a supplementary argument for its systematic use in hospitalized patients.

Several algorithms of decisions, namely, the “Programme National Nutrition et Santé”, Malnutrition Universal Screening Tool [7], and Nutritional Risk Index [8], have been proposed in the strategy of nutritional support. None of these algorithms led to a rational proposal of the adequate nutritional mixture consistent with all the parameters characterizing the patients. The proposed software could be considered as a new step in rationalization and optimization of nutritional strategies in hospitalized patients, in reference to the current knowledge in this field.

In all hospital departments, computerized systems that systematically detect malnutrition in hospitalized patients could offer the possibility of adequate nutritional support together with corresponding statistical and prospective studies.

## 5. Conclusions

Regarding its frequency and its medical and economic consequences, malnutrition in hospitalized patients has been the object of numerous studies. Nevertheless, in 2012, malnutrition often remains unknown/misdiagnosed in hospitalized patients and subsequently undertreated if not untreated. This could be prevented by adequate nutritional support strategies, coming from modern techniques, including computerized programs.

## Figures and Tables

**Figure 1:**
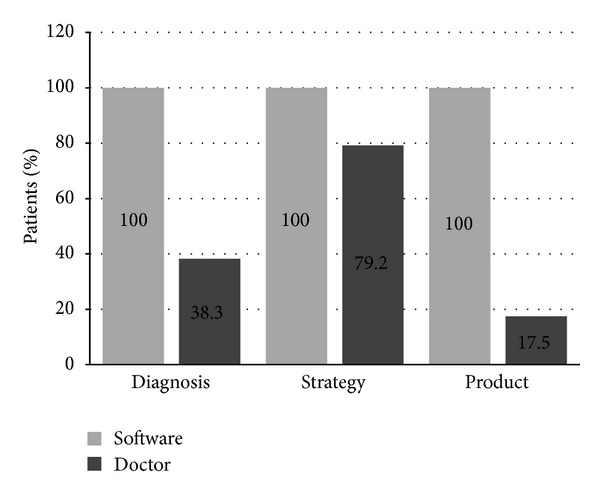
Relationship between software and doctors. Diagnosis: evaluation of malnutrition; strategy: strategy of nutritional support; product: nutritional mixture.

**Table 1:** Patients included in the study.

HIV infection	1 (0,8%)
Anorexia	1 (0,8%)
Rectum-colon cancer	40 (33,3%)
Pancreas cancer	4 (3,3%)
Gastric cancer	12 (10%)
Liver cancer	2 (1,7%)
Peritoneal cancer involvement	2 (1,7%)
Short bowel syndrome <1 m	6 (5%)
Malabsorption	5 (4,2%)
Chronic inflammatory bowel disease	7 (5,8%)
Chronic pancreatitis	2 (1,7%)
Gastrointestinal extended lesions	7 (5,8%)
Chronic intestinal pseudo-obstruction	4 (3,3%)
Other	27 (22,5%)

Total	120
